# Evaluating completion rates of COVID-19 contact tracing surveys in New York City

**DOI:** 10.1186/s12889-024-17920-4

**Published:** 2024-02-09

**Authors:** Kaiyu He, Steffen Foerster, Neil M. Vora, Kathleen Blaney, Chris Keeley, Lisa Hendricks, Jay K. Varma, Theodore Long, Jeffrey Shaman, Sen Pei

**Affiliations:** 1 Department of Biostatistics, Mailman School of Public Health, Columbia University, New York, NY 10032 USA; 2 New York City Department of Health and Mental Hygiene (DOHMH), Long Island City, NY 11001 USA; 3 NYC Health + Hospitals, New York, NY USA; 4 Department of Population Health Sciences, Weill Cornell Medical College, New York, NY 10065 USA; 5 Department of Population Health, New York University, New York, NY 10016 USA; 6 Department of Environmental Health Sciences, Mailman School of Public Health, Columbia University, New York, NY 10032 USA; 7 Columbia Climate School, Columbia University, New York, NY 10025 USA

**Keywords:** COVID-19, Contact tracing surveys, Survey completion rates, Random forest algorithm, Log-binomial regression, Model prediction

## Abstract

**Importance:**

Contact tracing is the process of identifying people who have recently been in contact with someone diagnosed with an infectious disease. During an outbreak, data collected from contact tracing can inform interventions to reduce the spread of infectious diseases. Understanding factors associated with completion rates of contact tracing surveys can help design improved interview protocols for ongoing and future programs.

**Objective:**

To identify factors associated with completion rates of COVID-19 contact tracing surveys in New York City (NYC) and evaluate the utility of a predictive model to improve completion rates, we analyze laboratory-confirmed and probable COVID-19 cases and their self-reported contacts in NYC from October 1st 2020 to May 10th 2021.

**Methods:**

We analyzed 742,807 case investigation calls made during the study period. Using a log-binomial regression model, we examined the impact of age, time of day of phone call, and zip code-level demographic and socioeconomic factors on interview completion rates. We further developed a random forest model to predict the best phone call time and performed a counterfactual analysis to evaluate the change of completion rates if the predicative model were used.

**Results:**

The percentage of contact tracing surveys that were completed was 79.4%, with substantial variations across ZIP code areas. Using a log-binomial regression model, we found that the age of index case (an individual who has tested positive through PCR or antigen testing and is thus subjected to a case investigation) had a significant effect on the completion of case investigation – compared with young adults (the reference group,24 years old < age <  = 65 years old), the completion rate for seniors (age > 65 years old) were lower by 12.1% (95%CI: 11.1% – 13.3%), and the completion rate for youth group (age <  = 24 years old) were lower by 1.6% (95%CI: 0.6% –2.6%). In addition, phone calls made from 6 to 9 pm had a 4.1% (95% CI: 1.8% – 6.3%) higher completion rate compared with the reference group of phone calls attempted from 12 and 3 pm. We further used a random forest algorithm to assess its potential utility for selecting the time of day of phone call. In counterfactual simulations, the overall completion rate in NYC was marginally improved by 1.2%; however, certain ZIP code areas had improvements up to 7.8%.

**Conclusion:**

These findings suggest that age and time of day of phone call were associated with completion rates of case investigations. It is possible to develop predictive models to estimate better phone call time for improving completion rates in certain communities.

**Supplementary Information:**

The online version contains supplementary material available at 10.1186/s12889-024-17920-4.

## Introduction

Contact tracing, the process of identifying people who have recently been in contact with someone diagnosed with an infectious disease, is widely used to inform interventions that reduce the spread of infectious diseases. During the acute phase of the COVID-19 pandemic, contact tracing was used in many countries [1–6] and jurisdictions in the United States (US) [7–9]. Data collected from such contact tracing efforts supported characterization of the epidemiological properties of SARS-CoV-2 [10–12] and community transmission patterns of the virus [13, 14]. In addition to improving scientific understanding of SARS-CoV-2, modeling studies indicate that contact tracing substantially reduces transmission of SARS-CoV-2 [15–21]. Recent studies estimated that case investigation and contact tracing in the US have reduced transmission 0.4% – 32% in 14 US jurisdictions from June through October 2020 [22] and averted 1.3% – 65.8% of the cases not prevented by vaccination and other nonpharmaceutical interventions from November 2020 to January 2021 [23].

The citywide contact tracing program (“Trace”) in New York City (NYC), part of the NYC Test & Trace Corps [24], was launched on June 1, 2020. This initiative aimed to provide contact tracing, testing, and resources to support isolation and quarantine (for residents not up to date on vaccinations after vaccines were available) and limit morbidity and mortality from COVID-19 in NYC. Three types of interactions were performed during the program. (1) *Case investigation*. Contact tracers made phone calls to confirmed cases and probable cases (defined as individuals with epidemiological linkage to confirmed cases and meeting clinical criteria such as acute onset or worsening of at least two of the following symptoms or signs: fever, chills, sore throat, diarrhea, fatigue, congestion or runny nose, etc. [25]) to perform a case investigation. Information about close contacts and places visited during the infectious period was elicited during the interview. (2) *Contact intake*. Contacts were called by contact tracers to notify them of their exposure status and were encouraged to quarantine and get tested. (3) *Monitoring*. Both cases and contacts were monitored daily through phone calls or text messages for the duration of their isolation or quarantine. A detailed description of the NYC case investigation and contact tracing operation is provided in Blaney et al. [8]

Case investigation included asking infected persons about the individuals and settings with which they were in contact during their infectious period. As a consequence, completion rates of case investigation interviews critically impact the success of contact tracing efforts. Understanding how NYC residents responded to case investigation calls and the key factors associated with higher completion rates can help design improved interview protocols for ongoing and future contact tracing programs.

We mainly made two contributions in this paper. Firstly, we used a log-binomial regression model to interpret the variables influencing the completion rates of COVID-19 contact tracing surveys in New York City. By examining the factors associated with the completion rates, we can glean insights into the demographic and behavioral characteristics that may facilitate or hinder the efficacy of these surveys. Second, we utilized a predictive model to enhance the methodology for increasing completion rates of these surveys in the future. The successful identification of influential factors and the subsequent application of a predictive model hold the promise of improving the effectiveness of phone-based contact tracing efforts, a cornerstone intervention in the management of infectious diseases.

## Methods

### Data

We analyzed 742,807 records of case investigation calls made from October 1st, 2020, through May 10th, 2021. In our study, confirmed cases include those identified through PCR or antigen testing. Furthermore, in alignment with the Council of State and Territorial Epidemiologists (CSTE) criteria [25], individuals who meet the specifications for a probable case are also considered in our case investigations. This approach ensures that our analysis encompasses a comprehensive range of COVID-19 cases, both confirmed and probable. These data were accessed on May 10th, 2021. Key case investigation information included the date of birth of index cases, ZIP code of home location, phone call time, and whether the phone interview was completed. Informed consent was obtained during the phone calls between contact tracers and participants prior to the collection of contact tracing information, which was documented in the contact tracing records. For minor participants, informed consent was obtained from parents or guardians. Use of this dataset in this study was approved by Columbia University Institutional Review Board (IRB) AAAT2182.

The initial phone call placed by the Trace team was recorded as “attempted”. If the index case answered the phone call, the interaction was recorded as “reached”. Phone calls were marked “completed” if all mandatory steps of interviews were completed. An interaction that was recorded as attempted or reached, but not completed, went back into the queue for a call attempt later that day. After three failed attempts to reach a person, the case was sent to a Special Investigations queue, where a community engagement team worked to reach the person either by phone, email, or in-person [26]. Before a home visit was attempted by a community engagement team, Information Gatherers searched other databases to see if additional contact information could be found. Note that case investigations for individuals younger than 18 years old were completed by parents or guardians. In addition, Trace did not conduct interviews on individuals living in nursing homes and long-term care facilities.

We used several variables at the ZIP code level for this analysis, including total population size, percentage of Black residents, percentage of Hispanic residents, median household annual income, percentage of residents with a bachelor’s degree, and mean household size. These covariates were selected to represent demographic and socioeconomic variations across NYC ZIP code areas. Data were compiled from the 5-year American Community Survey (ACS) [27]. We downloaded the 2020 estimates for these variables using the R package tidycensus (27) on May 10th, 2021.

### Regression model

For each case investigation, available information included age of the index case, ZIP code of home location, and time of day of phone call. To provide additional explanatory variables, we included several ZIP code-level characteristics. These ZIP code-level variables, although not necessarily reflecting the exact condition of each index case, represent possible demographic and socioeconomic status of the individual, which may differentiate the completion rate across ZIP code areas. We defined three age groups $(age\le 24\ years\ old\ (youth)$, $24\ years\  old< age\le 65\ years\ old\ (young\ adults)$, and $age > 65\ years\ old\ (seniors)$and four phone call time intervals $(9 am\le T<12 pm$, $12 pm\le T<3 pm$, $3 pm\le T<6 pm$, and $6 pm\le T\le 9 pm$). A log binomial regression model was fitted to the binary completion status for each case investigation $i$, controlling for demographic and socioeconomic conditions in ZIP code area ${l}_{i}$ where the index case resided. Specifically, the model is described by the following equation:

$$log\left({p}_{i}\right)={\beta }_{0}+{\beta }_{1}\times \%Black\ resident\left({l}_{i}\right)+{\beta }_{2}\times \%Hispanic\ resident\left({l}_{i}\right)+ {\beta }_{3}\times median\ household\ income\left({l}_{i}\right)+{\beta }_{4}\times \%bachelo{r}{\prime}s\ degree\left({l}_{i}\right)+{\beta }_{5}\times mean\ household\ size\left({l}_{i}\right)+{\beta }_{6}\times ag{e}_{senior\left(i\right)}+{\beta }_{7}\times ag{e}_{youth\left(i\right)}+{\beta }_{8}\times call\ time \left(9 am\le {T}_{i}<12 pm\right)+{\beta }_{9}\times call\ time \left(3 pm\le {T}_{i}<6 pm\right)+{\beta }_{10}\times call\ time \left(6 pm\le {T}_{i}\le 9 pm\right)+{\varepsilon }_{i}. (1)$$

Here ${p}_{i}$ is the completion probability for case investigation $i$, ${\beta }_{0}$ is the intercept, and ${\varepsilon }_{i}$ is the error term. Note that we used an implicit reference for phone call time – $12 pm\le T<3 pm$. Continuous explanatory variables were standardized (mean zero and variance one) before running the regression model to address the different scales of variables (e.g., percentage of population versus household income).

### Predictive model

While regression models are suitable for interpreting the effects of explanatory variable, they often have limitations in prediction in practical applications. We complemented our approach by experimenting with a random forest model. This decision was driven by the need for a more pragmatic and predictive tool, especially for forecasting optimal time of day of phone call. While the regression model provided valuable insights, it often recommended a limited range of call time. Experimenting with several machine learning approaches, we found that the random forest model performed well in identifying a broader and more evenly distributed range of time of day of phone call, capturing the non-linear intricacies of our data more effectively.

We used a random forest model [28] to predict the highest completion rate for case investigation as a function of time of day of phone call. Due to the limited availability of individual-level variables, predicting the completion status for each case investigation is challenging. We therefore switched the prediction target to the average completion rate for case investigations conducted for a certain age group within a time interval in each ZIP code area. We defined three age groups ($age\le 24\ years\ old$, $24\ years\ old < age\le 65\ years\ old$, and $age>65\ years\ old$) and four call time intervals ($9 am\le T<12 pm$, $12 pm\le T<3 pm$, $3 pm\le T<6 pm$, and $6 pm\le T\le 9 pm$). The prediction target was set as the average completion rates in all ZIP-age-call time groups, ${y}_{zip,age,call time}$. In addition to age groups and call time intervals, we included the ZIP code-level demographic and socioeconomic variables in Eq. (1) for index cases as predictors in the random forest model.

We randomly selected 80% of case investigation calls as training data and held the remaining 20% for out-of-sample validation. Using the selected 80% of records, we trained the random forest model using a tenfold cross-validation with the objective to minimize the RMSE (root-mean-square error) for mean completion rates. The optimized random forest consisted of 500 decision trees, each with one randomly selected predictor. Nodes in decision trees were split using the rule of variance (i.e., choosing the cut-point of predictor values that minimized the sum of the variances of split samples) under the constraint that each newly created node contained at least five samples. Other machine learning approaches such as regression tree and elastic net linear regression were also tested. The random forest model exhibited superior performance with a similar RMSE but a lower variation in terms of prediction error (i.e., more robust predictions). As a result, we presented the results from the random forest model as the main findings.

We quantified the importance of each variable in the random forest model by examining the degradation of prediction accuracy, measured by RMSE, after the variable was randomly permuted among all training data. We performed 20 independent permutations (Monte Carlo replications) and obtained the distributions of degradation – the permutation of an important variable would lead to a larger increase of RMSE.

### Counterfactual experiment

We conducted a counterfactual experiment using the random forecast model to evaluate the potential improvement in completion rate. We divided the data into training and testing sets. 80% of case investigation calls were used as training data and the remaining 20% were held for out-of-sample validation. First, we trained a random forest model using the training data. Then, for the test data, we created permutations with 4 time slots, meaning each individual was replicated 4 times with different time slots. This resulted in 3 counterfactual rows for each individual call. We used our trained random forest model to predict the completion rate for these counterfactual rows. Consequently, each row received a model-predicted completion rate. We treated the call time with the highest model-predicted completion rate as the model-predicted best call time. We then categorized the real-world test data (dropping all added counterfactual rows) into two groups: The first group consisted of individuals whose actual call time matched the predicted time slots. The second group comprised individuals whose actual call time did not match the predicted time slots. We calculated the completion rate for each group and compared the results from the two groups.

## Result

### Basic statistics of case investigation

**Fig. 1:**
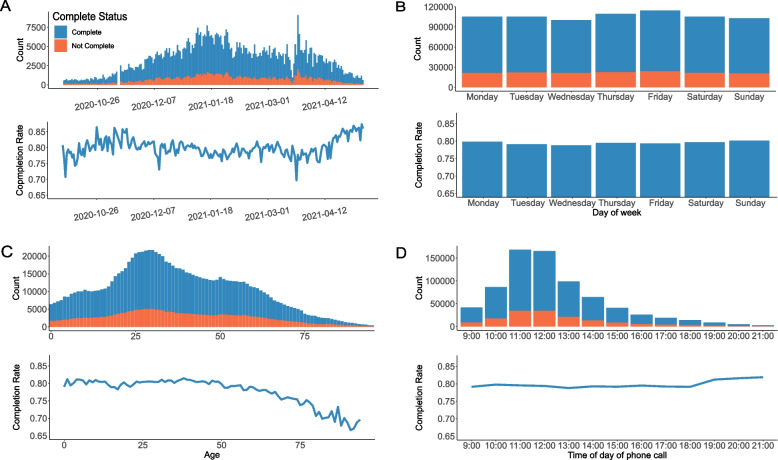
Key statistics of case investigation phone calls and completion rates in NYC: **A** The daily number of phone calls (upper) and completion rates (lower) during the study period. **B** The total number of phone calls (upper) and completion rates (lower) on each day of week. **C** The number of phone calls (upper) and completion rates (lower) for individuals of different ages. **D** The number of phone calls (upper) and completion rates (lower) for different phone call times

**Fig. 2:**
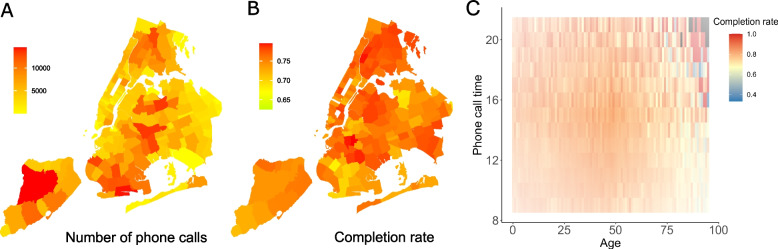
The geographical distribution of case investigation phone calls and completion rates in NYC: **A** The total number of phone calls in each ZIP code area. **B** The average completion rates in each ZIP code area. **C** Completion rate as a function of index case age and the phone call time (from 9 am to 9 pm)

From October 1st 2020 to May 10th 2021, 89% of case investigation calls reached index cases and 79.4% were completed. The daily number of phone calls for case investigation mirrored the trend of confirmed cases within the study period. The daily completion rate remained relatively stable over time, fluctuating between 75 and 85% (Fig. 1A). The total number of phone calls made on weekdays and weekends were similar with almost the same average completion rate (Fig. 1B). Young adults aged 20 to 49 years old constituted most index cases (Fig. 1C). On average, older index cases had a lower completion rate (Fig. 1C). The largest number of phone calls was made between 11 am and 12 pm (Fig. 1D). Phone calls made from 7 to 9 pm had the highest mean completion rate (Fig. 1D). The total number of case investigation phone calls varied across NYC ZIP code areas (Fig. 2A). Similarly, the completion rate exhibited considerable variation across different ZIP codes, ranging from 69.3% to 87.3% (Fig. 2B). We further found that the completion rate varied across the age of index cases and the phone call time (Fig. 2C) – people over 65 years old were less likely to complete case investigation before 12 pm.

### Factors associated with completion rates

Based on our regression model (Table 1), a higher percentage of Black residents or Hispanic residents were associated with higher completion rate. A 10% increase of % Black residents and % Hispanic residents in ZIP code areas were associated with a 1.6% (95%CI: 1.4% – 3.2%) and 3.4% (95%CI: 3.1% – 3.6%) higher completion rate, respectively. Other variables being equal, median household income in ZIP code areas was positively associated with the prevalence ratios of completing surveys. A $10,000 increase of median household income was associated with a 1.4% (95%CI: 1.1% – 1.7%) higher completion rate. An increase of 1 person of average household size in ZIP code areas was associated with a 2.1% (95%CI: 0.5% – 3.6%) lower completion rate. Notably, index case age had a significant effect on the completion rate of case investigation – compared with young adults (the reference group, $$24 years old<age\le 65 years old$$), the completion rate for seniors ($$age>65 years old$$) were lower by 12.1% (95%CI: 11.1% – 13.3%), and the completion rate for youth group ($$age\le 24 years old$$) were lower by 1.6% (95%CI: 0.6% –2.6%). In addition, phone calls made from 6 to 9 pm had a 4.1% (95% CI: 1.8% – 6.3%) higher completion rate compared with phone calls attempted from 12 and 3 pm. Other variables were not statistically significant. We also fitted a log-binomial model with an interaction term between phone call time and age group. The estimated coefficients are provided in Table A1 in Supplementary Materials. For seniors, phone calls made at 3 pm – 6 pm and 6 pm – 9 pm had a 11.6% (95% CI: 6.6% – 16.9%) and 9.3% (95% CI: 1.8% – 17.3%) higher completion rate compared with those made at 12 pm – 3 pm (the reference group). The completion rate of phone calls made at 9 am – 12 am for seniors was not significantly different from those made at 12 pm – 3 pm (the reference group).

**Table 1:** Estimated prevalence ratios in the log-binomial regression model. For categorical variables, we used Age (Young adult) and Call time (12 pm-3 pm) as the reference (i.e., their prevalence ratio is 1). The prevalence ratios were rescaled to represent when each variable increases by 1 unit (the last column), the relative change in the completion rate

Variables	Prevalence Ratio	95% CI	*P*-Value	Unit
%Black resident	1.016	(1.014, 1.032)	< 0.0001	10%
%Hispanic resident	1.034	(1.031, 1.036)	< 0.0001	10%
Household income	1.014	(1.011, 1.017)	< 0.0001	$10,000
%Bachelor	1.006	(0.991, 1.022)	0.445	10%
Household size	0.979	(0.964, 0.995)	0.010	1 person
Age (Senior)	0.879	(0.867, 0.889)	< 0.0001	NA
Age (Young Adult)	Reference	Reference		
Age (Youth)	0.984	(0.974, 0.994)	0.0015	NA
Call time (3 pm-6 pm)	1.002	(0.988, 1.015)	0.802	NA
Call time (6 pm-9 pm)	1.041	(1.018, 1.063)	0.0002	NA
Call time (12 pm-3 pm)	Reference	Reference		
Call time (9am-12 pm)	1.023	(1.014, 1.032)	< 0.0001	NA

### Improving completion rate using machine learning

**Fig. 3:**
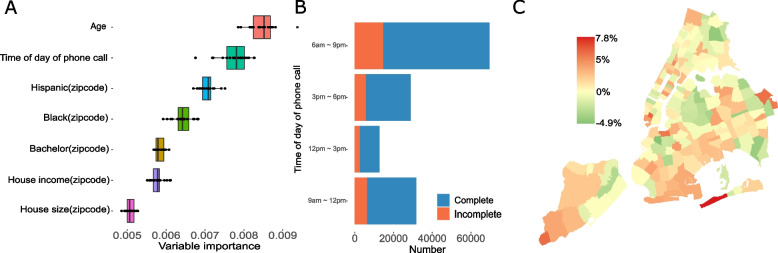
Evaluation of a random forecast model for predicting the best day of time of phone call **A** Ranking of the importance of variables in the random forest model. Age and time of day of phone call are individual-level variables and others are ZIP code-level variables. Importance is computed as the increase of RMSE when the focal variable is randomly permuted in prediction. The distributions were obtained from 20 independent permutations. **B** The predicted number of phone calls for each time interval using the random forest model. Outcomes (complete or not complete) were obtained from the real-world records in the validation data. **C** The change of completion rate using the best phone call time predicted by the random forest model in each ZIP code area. Positive values represent improved completion rate

We performed an initial evaluation on whether machine learning methods could be used to improve completion rates by optimizing the time of day of phone call. We trained a random forest model to predict the completion rate for phone calls using index case age, call time, and demographic and socioeconomic variables for the residential ZIP code area of the index case (see Methods). The mean RMSE in the out-of-sample validation is 0.066 (95% CI: 0.064 – 0.069). Age and time of day of phone call were found to be the two most important variables in prediction (Fig. 3A), consistent with the results in the regression model.

Using the 20% of case investigation records held for out-of-sample validation, we applied the random forest model to estimate the best time of day of phone call for case investigation with the highest expected completion rate. The distribution of the estimated best call time is shown in Fig. 3B. A large proportion of phone calls were directed to the evening time from 6 to 9 pm, in line with the findings from the regression model. On average, the expected mean completion rate in NYC computed using the random forest model increased by 1.2% compared with the actual mean completion rate. This overall improvement is limited partly because the completion rate was already high before optimization and the effect of call time on completion rate is relatively small (Table 1). However, the improvement varied considerably across NYC ZIP code areas (Fig. 3C). The expected completion rate increased by up to 7.8% in certain ZIP code areas, while there were locations with no apparent improvement or even decreased completion rates. Although overall we have a better completion rate after using the predictive model, some areas did get a lower completion rate. This counterfactual experiment indicates that the random forest model can be potentially useful in certain ZIP code areas for improving the completion rate of case investigation.

## Discussion

In this study, we examined the completion rates of COVID-19 contact tracing surveys in New York City during October 1st 2020 and May 10th 2021. We observed substantial variation of case investigation completion rates across ZIP code areas and performed statistical analyses to understand the factors associated with this variation. We found that, while the overall completion rate was high in NYC, senior residents were relatively less likely to complete the surveys, especially calls before 3 pm. Interestingly, survey phone calls made during evening time (6 pm – 9 pm) were more likely to be completed, possibly because people may not be able to answer survey phone calls during daytime hours. We further used a random forest model to assess its potential utility to predict the best phone call time for improving completion rates. While the overall improvement was limited, we found the random forest model was able to improve the expected completion rate by up to 7.8% in certain NYC ZIP code areas.

Our findings have direct implications on operations of phone call surveys. We found difference in completion rates depending on the age of index cases and attempted phone call time. Leveraging this difference, it might be possible to select attempted phone call time to improve survey completion rates. For instance, for persons aged over 65 years old, phone calls should be avoided before 3 pm (Table A1). It would be useful to evaluate the benefit of directing phone calls to the afternoon after 3 pm and evening hours. Phone call center was open 9 am to 9 pm; shift changes may be required to update work schedules. Given the potential utility of machine learning models, how to use these tools to support the deployment of resources in real-world settings should be explored. In addition, whether it is possible or ethical to gather more information to improve the performance of predictive models should be discussed. Besides, during the counterfactual experiment, although we observed an overall better completion rate after employing the predictive model on a larger scale, some areas experienced a lower completion rate. This might be because the model's predictions were less accurate in these specific regions, possibly due to variations in local demographics, cultural factors, or differences in phone usage patterns. To better understand these regional discrepancies, we need more detailed data, like local work schedules and phone call patterns.

A few limitations exist in this work. First, limited by data availability, only a few individual-level variables (age, phone call time, home locations) were used in the statistical analysis and the predictive model. Should additional individual-level variables become available, the performance of the predictive model might be further improved. Second, the results from the predictive model do not necessarily reflect real-world outcomes when the predictive model is used in practice. Interpretation of these results should therefore be made cautiously. Third, we were unable to tease out the effect of Community Engagement Specialists (CES) as interviews completed by a CES would be recorded the same as a phone call. We were also unable to analyze the effect of Contact Tracers who made phone calls on completion rates.

Telephone surveys are an important means of data collection, including for surveys on health conditions and resources in local communities associated with health-related services. The finding that survey phone calls made at a given time in certain communities yielded better response may have a broader implication in those settings. Our analysis suggests that surveys conducted through phone calls should be tailored to particular communities to improve completion rates and save resources.

### Supplementary Information

**Additional file 1.**

## Data Availability

Demographic and socioeconomic data for NYC zip code tabulation areas (ZCTA) were compiled from the 5-year American Community Survey (ACS) (https://www.census.gov/programs-surveys/acs/data.html). We downloaded the 2020 estimates for these variables using the R package tidycensus. Contact tracing records and individual testing results are subject to restrictions for the protection of patient privacy. Requests for data access should be addressed to NYC DOHMH and NYC Health + Hospitals or the corresponding author. The corresponding author will facilitate communications with NYC DOHMH and NYC Health + Hospitals, who will provide details of any restrictions imposed on data use via data use agreements.
