# Supplementary material for: Evaluating completion rates of COVID-19 contact tracing surveys in New York City
Source: BMC Public Health. 2024 Feb 9;24:414. doi: 10.1186/s12889-024-17920-4 (PMC10854191; doi:10.1186/s12889-024-17920-4)
Supplement: Supplementary file 1 — Additional file 1. [file 12889_2024_17920_MOESM1_ESM.docx]

**Evaluating completion rates of COVID-19 contact tracing surveys in New York City**

**Table A1. Estimated prevalence ratios in the log-binomial regression model with interaction terms.** The prevalence ratios were rescaled to represent when each variable increases by 1 unit (the last column), the relative change in the completion rate.

| **Variables** | **Prevalence Ratio** | **95% CI** | **P-Value** | **Unit** |
| --- | --- | --- | --- | --- |
| **%Black resident** | 1.016 | (1.014, 1.018) | <0.0001 | 10% |
| **%Hispanic resident** | 1.034 | (1.031, 1.036) | <0.0001 | 10% |
| **Household income** | 1.014 | (1.011, 1.017) | <0.0001 | $10,000 |
| **%Bachelor** | 1.006 | (0.991, 1.022) | 0.444 | 10% |
| **Household size** | 0.979 | (0.964, 0.995) | 0.010 | 1 person |
| **Age (Senior)** | 0.86 | (0.84, 0.88) | <0.0001 | NA |
| **Age (Young Adult)** | Reference | Reference |  |  |
| **Age (Youth)** | 0.971 | (0.956, 0.986) | 0.0002 | NA |
| **Call time**  **(3pm-6pm)** | 0.98 | (0.964, 0.996) | 0.0162 | NA |
| **Call time**  **(6pm-12pm)** | 1.02 | (0.995, 1.047) | 0.138 | NA |
| **Call time**  **(12pm-3pm)** | Reference | Reference |  |  |
| **Call time**  **(9am-12pm)** | 1.02 | (1.009, 1.031) | <0.0004 | NA |
| **Call time**  **(3pm-6pm)**  **&Age(Seniors)** | 1.116 | (1.066, 1.169) | <0.0001 | NA |
| **Call time**  **(6pm-9pm)**  **&Age(Seniors)** | 1.093 | (1.018 1.173) | 0.0143 | NA |
| **Call time**  **(9am-12pm)**  **&Age(Seniors)** | 1.016 | (0.987, 1.046) | 0.2866 | NA |
| **Call time**  **(3pm-6pm)**  **&Age(Youth)** | 1.055 | (1.021, 1.091) | 0.0013 | NA |
| **Call time**  **(6pm-9pm)**  **&Age(Youth)** | 1.048 | (0.998, 1.1) | 0.0628 | NA |
| **Call time**  **(9am-12pm)**  **&Age(Youth)** | 1.012 | (0.99, 1.035) | 0.2858 | NA |
